# Loss of live coral compromises predator-avoidance behaviour in coral reef damselfish

**DOI:** 10.1038/s41598-018-26090-4

**Published:** 2018-05-17

**Authors:** Lisa Boström-Einarsson, Mary C. Bonin, Philip L. Munday, Geoffrey P. Jones

**Affiliations:** 1 ARC Centre of Excellence for Coral Reef Studies, James Cook University, Townsville, Australia; 2 College of Marine and Environmental Sciences, James Cook University, Townsville, Australia

## Abstract

Tropical reefs have experienced an unprecedented loss of live coral in the past few decades and the biodiversity of coral-dependent species is under threat. Many reef fish species decline in abundance as coral cover is lost, yet the mechanisms responsible for these losses are largely unknown. A commonly hypothesised cause of fish decline is the loss of shelter space between branches as dead corals become overgrown by algae. Here we tested this hypothesis by quantifying changes in predator-avoidance behaviour of a common damselfish, *Pomacentrus moluccensis*, before and after the death of their coral colony. Groups of *P*. *moluccensis* were placed on either healthy or degraded coral colonies, startled using a visual stimulus and their sheltering responses compared over a 7-week period. *P*. *moluccensis* stopped sheltering amongst the coral branches immediately following the death of the coral, despite the presence of ample shelter space. Instead, most individuals swam away from the dead coral, potentially increasing their exposure to predators. It appears that the presence of live coral rather than shelter *per se* is the necessary cue that elicits the appropriate behavioural response to potential predators. The disruption of this link poses an immediate threat to coral-associated fishes on degrading reefs.

## Introduction

Habitat loss is the primary cause in population declines and loss of biodiversity in most ecosystems^1^. Population declines can occur as a direct demographic response to the loss of critical resources, such as food or shelter. However, animal responses to altered environments are often first evident from changes in behaviour^2^. Appropriate behavioural responses can also contribute to a species’ tolerance to, or recovery from, environmental change^3^. Behaviour can ameliorate the effects of habitat degradation provided species have the capacity to respond in a way that improves their chances of survival. Unfortunately, an increasing number of studies have demonstrated that habitat loss and degradation alter key signals or cues used by animals in vital decision-making, such as finding food and avoiding predators^4–6^. The links between these short-term behavioural responses and longer-term demographic responses must be established in order to understand and mitigate against the threats posed by increasing habitat loss across the planet.

The precise mechanisms by which a species responds to habitat loss may be related to the disruption of behavioural cues, the loss of habitat *per se*, or a combination of these factors. In extreme scenarios, complete habitat destruction leads to the loss of both the cue and the habitat simultaneously, which should enable the animal to make accurate decisions about alternate habitats^7^. However, anthropogenic disturbance can also lead to situations where altered or introduced environments mimic cues emitted by natural environments, causing a maladaptive behavioural response (‘ecological and evolutionary traps’)^4,8^. For example, many bird species are attracted to nest on agricultural pastures because they are structurally similar to native grasslands, but are then subject to low survival rates due to farm practices^4,9^. Habitat degradation can also lead to alteration or loss of cues associated with the habitat, without necessarily altering the quality of the resource provided by the habitat. In this case, an animal might choose not to use a suitable habitat simply because it does not possess the cues identifying it as appropriate habitat. While this “undervalued resource” scenario has been predicted theoretically^10^, it has rarely been demonstrated in a natural system. Despite the ubiquity of habitat degradation in global ecosystems, few studies have established mechanisms underpinning the threat to species, whether they are the direct effects of habitat loss on vital resources, a disruption of appropriate behavioural responses, or both.

Coral reef ecosystems have been experiencing an unprecedented loss of hard-corals, with the first decade of the 2000’s recording an average 20% decline in live coral cover on reefs worldwide^11–14^. Recent mass coral bleaching events in 2016 and 2017 suggests that this pattern of live coral loss is likely to continue^15,16^. Live corals provide the majority of structural complexity on coral reefs, and degradation of these biogenic habitats may negatively impact both the behaviour^17–19^ and abundance^20–22^ of coral reef fishes. Typically there is an almost immediate decrease in the abundance of obligate coral-associated species, specifically coral-feeding^23,24^ and coral-dwelling fishes^25^, when live coral declines. For species that rely solely on live coral tissue as a food source, like many butterflyfishes (Chaetodontidae), declines in abundance appear to result from loss of food resources as the coral tissue dies^26–28^. However, for most other coral-dwelling fishes, it is unknown whether these directly reflect increased mortality associated with declining resources, behavioural responses that follow from changing environmental cues, or a combination of these factors.

One hypothesis for the decline in coral-dwelling reef fish following habitat degradation is the loss of shelter spaces among coral branches, which would leave these fish more vulnerable to predators^29–31^. Live corals, especially structurally complex species, provide critical shelter space from predators; however habitat disturbances such as coral bleaching, crown-of-thorns starfish outbreaks and poor water quality degrade the quality of this shelter. Initially when the living coral tissue is lost, there is little change to the shelter available to coral-dwellers. However, in the weeks to months following tissue loss, structural degradation begins to occur through the settling of algae, sponges and invertebrates that overgrow the coral skeleton and reduce shelter spaces available between the branches. Eventually borers and grazers will contribute to the breakdown of the coral skeleton into rubble^32^. Given the importance of live coral as a shelter site from predators, it is not surprising that the effects of competitive interactions are exacerbated following coral mortality^18,33,34^. For example, coral-dwelling reef fish compete for access to refuge between the branches, and suffer increased mortality through predation when this resource is limited^35,36^. Consequently, declines in shelter space due to colony overgrowth by algae and invertebrates may increase predator-induced mortality and exacerbate competition for shelter, explaining the observed declines in coral-dwellers soon after disturbances that cause live coral loss.

There is increasing evidence that the presence of live coral provides an important cue to inform behavioural decisions about habitat suitability, which may be disrupted as soon as the coral dies. The loss of live coral itself can independently influence the behaviour of reef fishes, altering the strength of aggressive interactions^33,37^ and risk assessment^38^. Many reef fishes recruit primarily to live corals^20,39^ and adults often maintain a close association with living coral colonies. For example, the common reef fish *Chrysiptera arnazae* (previously *C*. *parasema*)^40^ migrated into remnant live portions of partially degraded coral colonies following habitat degradation^33^. This occurred immediately following the loss of the living coral tissue when shelter space had not yet been reduced. Similarly, another damselfish, *Pomacentrus amboinensis*, has been shown to vacate recently dead corals in search of healthy colonies nearby, but in contrast does not vacate colonies that are alive or bleached^41^. The mismatch between the timing of loss of coral tissue and loss of shelter provides an excellent setting for distinguishing between the roles of behavioural cues and shelter availability in ecological responses to habitat degradation.

**Figure 1:**
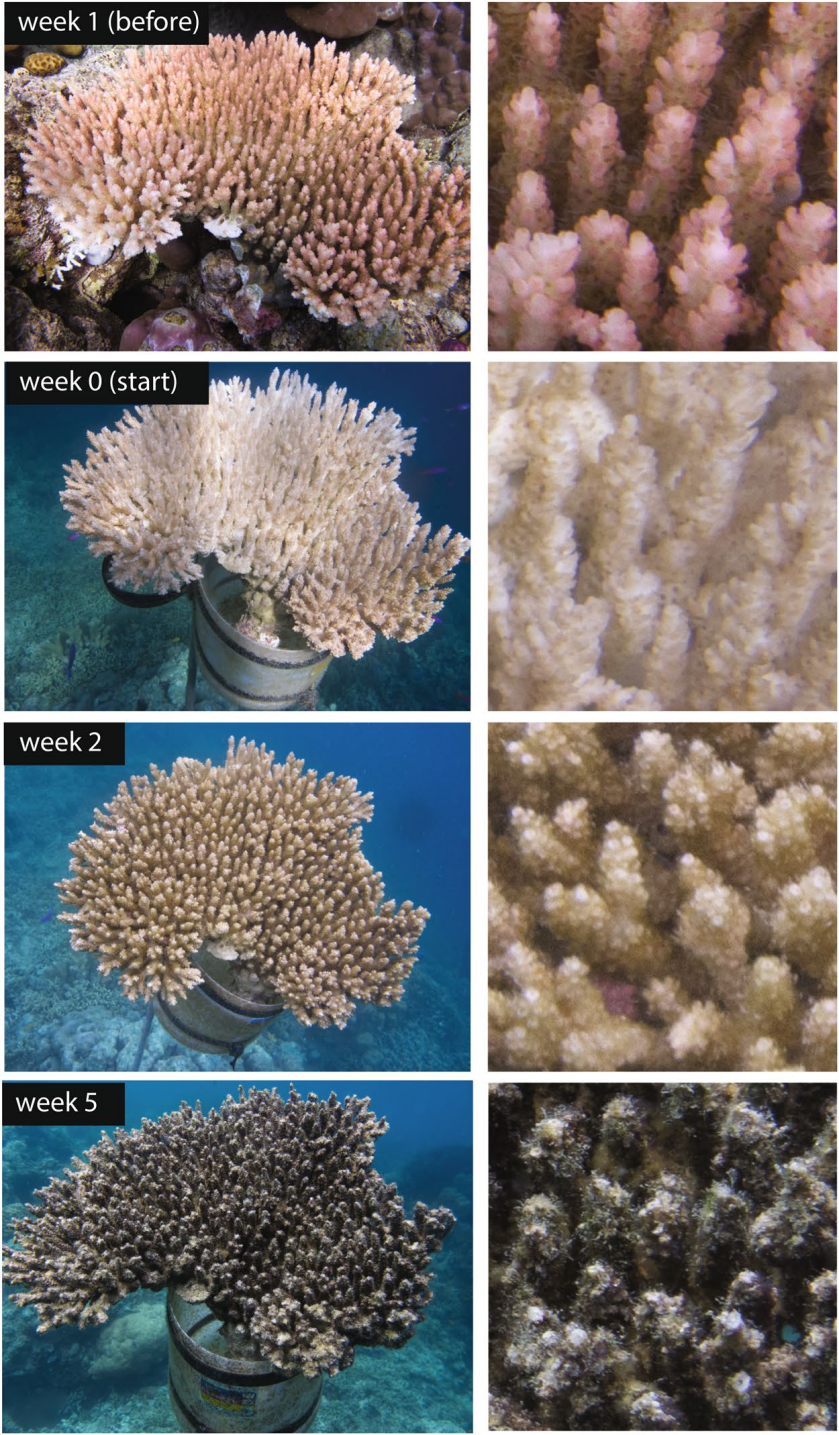
The progression of a degraded colony from live (week-1), immediately after degradation by COTS was complete (Week 0), followed by the accumulation of a thin layer of algae (week 2) through to a dense turf algae growing on the branches (Week 5). Right hand pictures depict an area approximately 50 mm × 50 mm (H × W). Note that colony is suspended in water column for photograph s only, and shelter behaviour of *P*. *moluccensis* was not tested in this position.

The aim of this study was to experimentally test whether the predator-avoidance behaviour of a common reef damselfish, *Pomacentrus moluccensis*, is affected by either the loss of coral tissue following the death of its host or the longer term loss of shelter space among coral branches. Specifically, we examined the links between the timing of changes in behaviour, loss of coral tissue and ultimate changes to shelter availability. First we analysed the level of association with habitat under ‘normal’ conditions by viewing video recordings of groups of fish on live and dead coral colonies. To test how this association changes when danger approaches we performed a startle experiment, where groups of *P*. *moluccensis* were placed on corals in different stages of degradation and then exposed to a darting visual stimulus. Their positions and responses to the visual stimulus were recorded using video cameras. Colonies were either live throughout the experiment (healthy) or suffered a complete loss of live coral tissue at the start of the testing period (degraded). The trials were conducted over the course of seven weeks to capture a gradient of habitat quality from healthy coral at the start, to dead but with shelter space unchanged (week 0, Fig. 1) and through a progressive overgrowth of the colony by the accumulation of algae and invertebrates (week 1–5 post-disturbance, Fig. 1). To test whether there were intercohort size-based differences in response to shelter, we included fish from two size groups; recently settled recruits (10–15 mm) and adults (>15–25 mm).

## Results

We tested the response of *P*. *moluccensis* to a visual startle device in cages placed on the reef. Groups of *P*. *moluccensis* were placed on live or degraded coral colonies (100% tissue consumed by crown-of-thorns starfish) inside a 90 × 90 cm mesh cage. A visual startle device was rapidly moved over the coral colony, and the response recorded using video cameras (Supplementary video S1). The trials continued for a total of 7 weeks (1 trial pre-degradation and 6 trials post-degradation).

### Pre-startle position

To evaluate the association with a coral colony prior to a threat approaching (the visual stimulus), we recorded the number of fish that were within a 5 cm distance of the coral colony immediately before a visual startle device entered the cage where fish were being held. A majority of *P*. *moluccensis* individuals were present on healthy colonies prior to the startle event, while association with the coral colony was more varied on degraded colonies (Figure S1). This is reflected in a significant interaction between the two main factors (colony type × trial week, Log likelihood chi-square 14.4, d.f. 6, p = 0.02).

### Sheltering position

**Figure 2:**
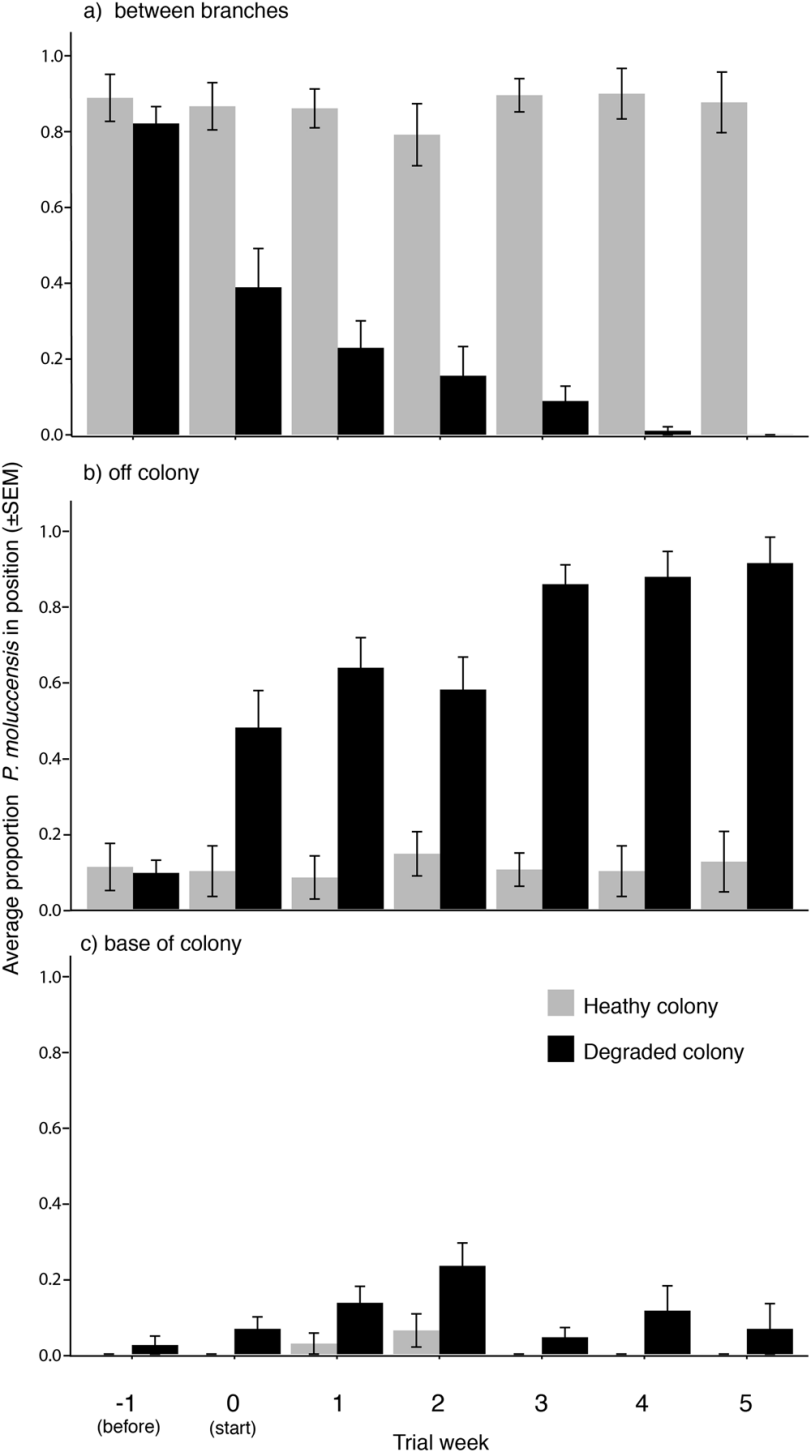
Average proportion of *Pomacentrus moluccensis* recorded either (**a**) sheltering between coral branches, (**b**) swimming off the coral colony (i.e not sheltering), and (**c**) at the base of the colony following a visual startle. Trials were conducted on either healthy (100% live) colonies (grey bars) or degraded colonies (black bars). Treatment colonies were 100% live in week -1, tissue recently dead (same day) in week 0, and with gradually accumulating algae and settling invertebrates in week 1–5. Each trial tested 6 *P*. *moluccensis* in a group, with 9 healthy and 15 degraded trials each week. Error bars indicates SEM.

When disturbed by a visual startle device, *P*. *moluccensis* were much more likely to shelter amongst the branches of healthy colonies, compared with degraded colonies (Fig. 2a, overall average proportion of *P*. *moluccensis* in branches of live colony ±0.02 SEM, dead colony 0.23 ± 0.03). While these effects were evident immediately following the degradation of the coral colony, these differences in behaviour between colony types also increased with time (Fig. 2a). There was a concomitant increase in individuals that exhibited no sheltering behaviour on degraded colonies (Fig. 2b, degraded colony 0.64 ± 0.04 SEM, healthy colony 0.12 ± 0.02). By contrast, there was no change in sheltering position on healthy colonies over the experimental period (Fig. 2a–c). Few individuals chose to shelter on the base of the colony in either healthy or degraded colonies (healthy colony 0.2 ± 0.06 SEM, degraded colony 0.10 ± 0.02).

A saturated log-linear model was the most appropriate model to compare the frequency of *P*. *moluccensis* in each category. Removal of the three-way interaction between all main factors (colony type, sheltering position and trial week) resulted in a significant increase in model deviance (Log likelihood chi-square 68.7, d.f. 12, p < 0.001). Therefore, an interaction among these factors influenced the frequency of *P*. *moluccensis* in each category.

A video of a typical trial and fish response can be viewed in supplementary online files (Supplementary video S1).

### Inter-cohort differences

There was no evidence of a size-based or intercohort difference in the fish that accessed prime shelter habitat amongst the branches of the coral colony (Log likelihood chi-square = 363.1, df = 299, p = 0.239, Figure S2).

## Discussion

This study has demonstrated that predator avoidance behaviour of *Pomacentrus moluccensis* is fundamentally and rapidly altered by coral mortality. Following the degradation of their colony, a majority of individuals stopped sheltering altogether and instead moved to high-risk locations off the colony when startled. In contrast, most fish on healthy colonies sheltered between live coral branches when startled. Given that behavioural changes occurred immediately following the degradation event, we argue that this is not due to declining shelter space available between degrading branches, but instead is a direct reaction to the loss of live coral tissue. The presence of live tissue appears to be necessary to elicit the coral-shelter seeking behavioural response, independently of any change in the quality of the shelter *per se*.

The disruption of sheltering behaviour suggests a potential mechanism underlying the loss of reef fishes following habitat degradation. The loss of live coral from coral bleaching, diseases and outbreaks of coral predators tend to leave the structure of the coral skeleton intact, only removing the living coral tissue. This initial degradation of the reef habitat is often followed by a rapid decline in fish species that either feed on live coral tissue^23,42,43^, live in obligate association with live corals^25,44^ or preferentially recruit into live corals^20^. Our results suggest that these responses are due the disruption of shelter seeking behaviour. Longer-term degradation of coral reefs occurs as space between the branches of coral colonies is lost within weeks of the degradation event, due to settling of algae and invertebrates. Ultimately, reefs may be reduced to rubble in a process expedited by invertebrate borers and storms over an estimated time-span of 4–10 years^45,46^. The loss of shelter and other resources are likely to account for a gradual decline in some coral-dwelling fishes, because refuges from predators are lost as the structural complexity of the coral habitat is lost^21,47,48^. Given that many studies fail to capture the time-period immediately following a disturbance event, this study offers a unique insight into mechanisms occurring prior to this loss of structural integrity. Our results are consistent with the idea that the loss of coral tissue represents the loss of an important behavioural cue, either for settlement or predator avoidance.

Our results are consistent with the concept of ‘undervalued resources’, where animals avoid high quality habitat because the environmental cues they use to assess habitat quality have been altered. Gilroy and Sutherland^10^ proposed almost a decade ago that this kind of scenario should be common, however evidence supporting this theory is lacking. The theory was proposed in response to the concepts of ‘ecological and evolutionary traps’ in which a cue prompts a behaviour that has been rendered maladaptive by environmental change. Prior to disturbances organisms respond to cues in a way that is adaptive in their natural environment^49^, but environmental change can decouple the cue-response relationship such that the response is now maladaptive^4,50^. In our case, the death of the habitat means the behavioural cue for sheltering within the habitat is missing, even though it would still be highly beneficial to do so to avoid predators. A dead coral habitat may not fit into the description of an ‘undervalued resource’ for very long, given the eventual loss of shelter over a few weeks; however, our result highlights the complex chain of events associated with the loss and degradation of habitats in ecological communities.

While the nature of the cue provided by corals may not be fully understood, our results suggests that the absence of live coral tissue fails to elicit the correct behavioural response to a potentially life threatening risk. This begs the question - which cue-response relationship, when disrupted, would cause individuals to swim past perfectly suitable habitat in favour of riskier locations and fail to shelter from predators? Currently, there are three potential mechanisms offered in the literature: (1) loss of camouflage from predators, (2) Changes to the visual perception of the habitat, and (3) changes to the olfactory perception of habitat.

A recently dead coral colony may leave reef fish vulnerable to predators as the bright white coral skeleton provides inadequate camouflage to the fish^17^. In this instance, the visual cue of a live-coral would signal a safe place to hide from predators. Indeed this has been demonstrated in aquaria where fish on bleached corals were twice as likely to be attacked by a predator compared to live corals^31^. However, this does not necessarily translate to the natural environment. In a study conducted during a natural degradation event, Bonin *et al*.^47^ found that fish on bleached corals did not suffer higher mortality than their counterparts on live corals. Similarly fish on live and bleached corals show the same level of fidelity to their coral habitat while individuals on dead corals are inclined to migrate in search for higher quality habitat^41^. Emigration increases vulnerability to predation as fish are forced to leave the shelter of coral hosts. Combined, these studies suggest that changes in behaviour in relation to dead coral habitat are not camouflage related, but rather responses to a cue used to identify suitable habitat.

Avoidance of dead coral may occur because it does not look like coral anymore to the fish, however no studies to date have directly investigated visual effects of habitat degradation to reef fishes. If the lack of pigmentation affects the visual assessment of dead coral colonies, then reactions should be similar between bleached and recently dead corals that have both lost their symbiotic algae and pigments^51^. In aquarium trials *P*. *moluccensis* selects live coral over bleached and dead coral when allowed to use visual cues only^52^; however such a cafeteria-style array may not be available in a reef environment. Indeed, during an ongoing bleaching event Bonin *et al*.^47^ did not record any difference in *P*. *moluccensis* recruitment or persistence on bleached and live colonies. This suggests that while fish favour live coral when given the choice in a side-by-side comparison, they are unlikely to detect a difference between live or bleached coral on the reef. Importantly, the literature suggests that this misidentification does not translate into a demographic consequence.

Reef fish behaviour is often mediated by olfactory stimuli such as the smell emitted by a predator and injured conspecifics^53–56^. The third hypothesis on why reef fish may avoid dead corals is that the chemical cocktail emitted by a dead coral may hinder the detection of predators^17^. Authors have argued that the smell of a dead coral colony, even when heavily diluted, is strong enough to obstruct alarm cues and cause congener *P*. *amboinensis* to cease predator-avoidance behaviour^38,57^. Following this argument, the different olfactory signal of dead coral colonies in this study could be enough to deter *P*. *moluccensis* from seeking refuge amongst the colony branches.

Finally, it is possible that the avoidance of dead coral was an attempt at locating a healthy coral colony nearby to shelter in. However, given the extreme level of habitat association exhibited in this fish species^58,59^, and the low likelihood that a severe crown-of-thorns outbreaks or bleaching event (two types of disturbances which cause the same degradation as that tested here) would leave sufficient remnant live colonies, we argue that this is unlikely to be a successful approach. Furthermore, remnant live coral colonies are likely to become crowded with high densities of conspecifics. *P*. *moluccensis* are vulnerable to density dependent interactions^60,61^, and are thus unlikely to benefit from relocating.

While we may not know the exact reason why the fish avoid dead coral colonies, our experiment suggests that they have lost the association with the habitat as a potential refuge site. A loss in association with degraded habitat has previously been described in *P*. *moluccensis*^39,52,62^ and for closely related damselfishes^33,63^; however this is the first time it has been demonstrated that this behaviour persists during predator evasion. While dead coral is not an entirely novel habitat for this species, our results demonstrate the strength and importance of *P*. *moluccensis* close association with living corals. In summary, this study has revealed the early disruption of predator-avoidance behaviour in reef fish following the death of branching corals, possibly leading to predation-induced mortality following habitat degradation. This is the first study to evaluate predator avoidance behaviour of reef fishes for a biological disturbance that eliminates living tissue but leaves habitat structure initially intact, such as crown-of-thorns outbreaks or coral bleaching. The early onset of behavioural change highlights the importance of live coral tissue itself, and how the loss of this tissue can fundamentally alter escape responses. The absence of predator avoidance behaviour offers a potential pathway to explain the immediate decline in coral-associated fish following habitat degradation. Our results highlight the consequences of anthropogenic disturbance on critical behavioural cues in the marine environment. The study adds to the growing body of work demonstrating that the disruption of natural cues by anthropogenic disturbances may be an important driver of changes to ecological processes important in maintaining marine biodiversity.

## Methods

### Study species

The lemon damsel, *Pomacentrus moluccensis*, is an obligate coral-dwelling damselfish (Pomacentridae) often found in dense aggregations on plating *Acropora* coral colonies on shallow reefs. They are known to retreat into the branches of their coral colony hosts to escape predators or other perceived threats (Beukers and Jones 1998). Given that access to shelter can be governed by differences in body size we size matched fish into groups of large and small fish to evaluate whether there is a intercohort difference in access to shelter. We collected *P*. *moluccensis* in two size classes: 10–15 mm (mean 13.4 mm ± 0.04 SEM), representing recently settled fish (<1 week post settlement) and 20–25 mm (mean 22.6 mm ± 0.07 SEM) representing young adults with a close association to individual coral colonies. Fish were collected using a dilute solution of clove oil anaesthetic^64^ and hand nets, and placed in a plastic bag. Individuals were allowed to recover from handling stress in the bag for a minimum of one hour with frequent water changes.

### Experimental protocols

We used an underwater “startle” experiment to test how predator avoidance behaviour of *P*. *moluccensis* was affected by the degradation of their coral habitat over a seven week period. Coral colonies were degraded and the behaviour of groups of *P*. *moluccensis* was recorded through time as colonies gradually accumulated algae and invertebrates. Thirty corymbose Acropora colonies were collected from the exposed reef crest of platform reefs in Kimbe Bay, Papua New Guinea (150°05′E, 5°25′S) and transported to the study reefs. Colonies were of similar size and branch morphology (mean size 754 cm^2^, ±32.9 SEM). Two cages were constructed of PVC pipes (90 cm W × H × D) covered in a thin mesh that allowed flow of water in and out of the cage. The cages served multiple purposes by protecting the fish from predators and restricting external visual disturbances. Cages were placed on flat sandy bottom in ~3 m of water, approximately 1 meter apart. Two opposing sides had entry and exit holes for a startle device to pass through. A 20 cm black torpedo-shaped object was rapidly pulled through the cage to startle the fish. The shape and size of the object resembled a potential predator. Scuba divers pulling the startle device were placed >5 m away from the cage to avoid disturbing the fish following a habituation period.

Coral colonies were placed inside experimental cages on a small rubble base and groups of six *P*. *moluccensis*, (three small and three large) were placed on colonies. We tested behaviour in a group, rather than testing individual fish, because *P*. *moluccensis* is a highly aggregating species^65^, and are rarely observed as single individuals on reefs. Fish were allowed to habituate to their new surroundings for 20 minutes. First, to analyse habitat association we recorded the number of fish within a 5 cm distance of the coral colony immediately before the startle device entered the cage. The startle device would then be pulled through the cage once, and the fish removed from the cage. Each group of fish was only tested once to avoid learning from previous trials. Each startle trial was recorded using two video cameras (GoPro Hero 3) mounted directly above and to the side of the coral colony. Recordings were played back in slow motion and predator avoidance behaviour was recorded. Predator avoidance behaviour was scored in three mutually exclusive and exhaustive categories; (1) retreat amongst coral branches, (2) shelter on base of colony, and (3) swimming off the colony into the surrounding water (unprotected). The size and position of each fish was recorded when the startle object was immediately above the coral colony. The intercohort test investigates whether there are any differences in access to shelter between large and small fishes. However, because there was no reciprocal experiment where equal densities of a single size fish were startled we cannot exclude innate differences in behaviour between juvenile and adult fish.

After the first set of trials were conducted, 25 colonies were placed in cages on the reef containing multiple individuals of the corallivorous crown-of-thorns starfish *Acanthaster* cf. *solaris (*previously known as *A*. *planci*)^66^. The starfish were allowed to consume 100% of the live coral tissue of these degraded colonies (~3 days). The colonies were thereafter placed in a shallow (~1.5–2 m deep) reef flat area where they were allowed to accumulate algae and settling invertebrates for the duration of the experiment (Fig. 1). The remaining five colonies were placed in cages without *A*. cf. *solaris* for three days to control for handling stress and were thereafter placed in the same shallow location as degraded colonies. We recorded no mortality, injury or disease in healthy colonies throughout the experiment. Trials were thereafter conducted weekly for a total duration of 7 weeks (before degradation, day of degradation and week 1–5 post-degradation). 24 trials were conducted each week (9 trials with healthy colonies trials and 15 trials with degraded colonies), using a random subset of 5 degraded and 3 healthy colonies. Each colony was used for three sequential trials using a different group of fish.

Using a caged experiment was necessary to provide a fair comparison of the two coral treatments, however we acknowledge that testing behavior in a cage (albeit on the reef) may change the behavior of fish. Despite this, given the aggregative nature and relatively small home range of *P*. *moluccensis* we feel confident that behaviors recorded here are similar to those occurring on the reef. We have included a video recording of a trial in the supplementary online material, including a failed trial in which a real predator (Moon wrasse, *Thallassoma lunare*) entered the cage. While these videos only depict single instances, they illustrate the difference in behavior between the two treatments, and the similarity in response to a genuine threat (Supplementary video S1).

### Statistical analysis

We tested whether the choice of predator avoidance behaviour (i.e. sheltering among the branches, on the base or off the colony) differed between healthy and degraded colonies using a log-linear analysis. The model included three factors: sheltering positions (3 categories: sheltering among the branches, on the base or off the colony), colony type (2 categories: healthy and degraded) and trial week (7 categories: before, start and week 1–5 post disturbance). The saturated model analyses the frequency (i.e. sum of *P*. *moluccensis* from each replicate trial) in each possible combination of the three factors and their interactions. Model selection was achieved by backwards elimination of terms in the model, and goodness-of-fit assessed using likelihood ratios. A significant chi-square-test between a reduced and full model indicates that the elimination of the excluded factor in the reduced model is not warranted. The most parsimonious model (i.e. the least complex model that best accounts for the variances in the observed frequencies) was then selected based on goodness-of-fit results. The use of a time factor does not necessitate a repeated measures analytical method given that a new group of fish were used during each trial event, and the tested colony in each trial was randomly selected from a larger set of possible colonies. No analyses violated assumptions of expected frequencies >5 in 80% and >1 in 100% of categories for log-linear analyses. All statistical analyses were conducted within the R-environment^67^. A second log-linear analysis was performed to evaluate whether habitat association differed between healthy and degraded colonies over the trial period. This test analysed whether the number of *P*. *moluccensis* present immediately prior to the startle event differed between colony types (2 categories: healthy and degraded colonies) over time (7 categories: categorical factor, 7 levels), and the interaction between the two main factors. Finally, a third log-linear analysis was performed to evaluate any size based differences in the number of fish with access to shelter space between the branches of the coral colony. The count of *P*. *moluccensis* sheltering amongst branches could then be evaluated for differences in body size (2 categories: large, small), trial week (7 categories) and colony type (2 categories: healthy and degraded).

Effect sizes were calculated for all analyses (sheltering position, size effects and numbers present pre-startle) as partial eta-squared (*η*^2^*p*) which can be interpreted as the unexplained variation in the dependent variable, plus the variation explained by the factor in question. This allows comparisons across different studies (i.e. meta-analyses), which may contain additional factors or covariates. Assumptions of independence and normality of residuals were examined visually. Tukeys HSD pairwise posthoc tests were used to investigate underlying patterns in the data.

### Ethics

The experiments described were approved under James Cook University Animal Ethics Committee permit A1899, in accordance with Queensland Animal Care and Protection Act 2001.

### Data availability

The datasets generated during and/or analysed during the current study are available from the corresponding author on request.

## Electronic supplementary material

Supplementary Video S1

Supplementary Figures S1 & S2
