# Supplementary material for: Loss of live coral compromises predator-avoidance behaviour in coral reef damselfish
Source: Sci Rep. 2018 May 17;8:7795. doi: 10.1038/s41598-018-26090-4 (PMC5958076; doi:10.1038/s41598-018-26090-4)
Supplement: Supplementary file 2 — Supplementary Figures S1 & S2 [file 41598_2018_26090_MOESM2_ESM.docx]

Loss of live coral compromises predator-avoidance behaviour in coral reef damselfish

**Author affiliations and contact information:**

Lisa Boström-Einarsson ^1,2^*, lisa.bostromeinarsson@my.jcu.edu.au

Mary C Bonin ^1,^ , mary.bonin@jcu.edu.au

Philip L Munday^1^, philip.munday@jcu.edu.au

Geoffrey P Jones^1,2^, geoffrey.jones@jcu.edu.au

^1^ARC Centre of Excellence for Coral Reef Studies, James Cook University, Townsville, Australia

^2^College of Marine and Environmental Sciences, James Cook University, Townsville, Australia

* Corresponding author


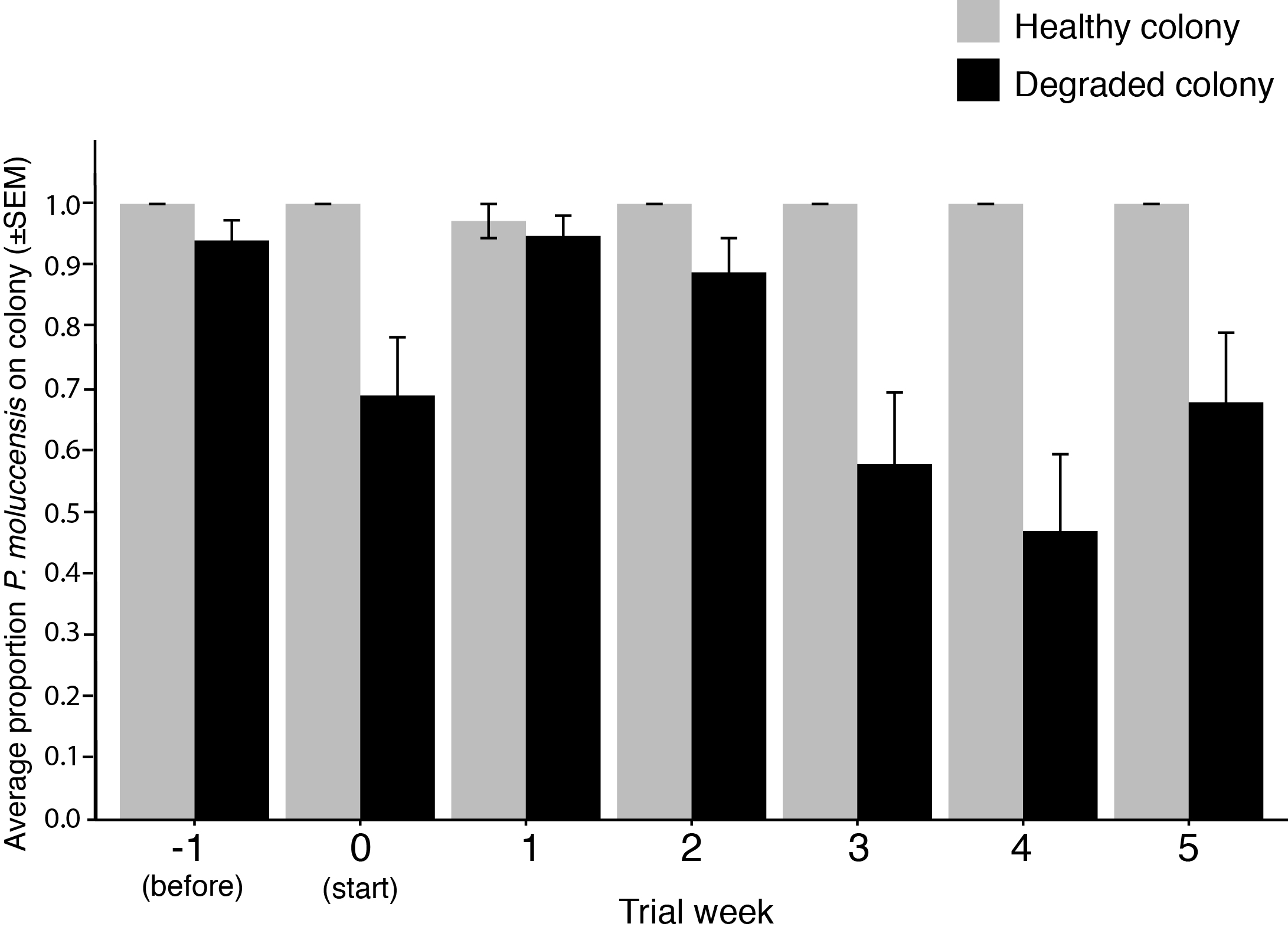


Supplemental Figure 1: The average proportion of *P. moluccensis* present on the control (grey bars) and treatment colonies (black bars) immediately before the startle event occurred over the 7 week trial period. Each trial tested 6 *P. moluccensis* in a group, with 9 control and 15 treatment trials each week. Error bars indicates SEM.


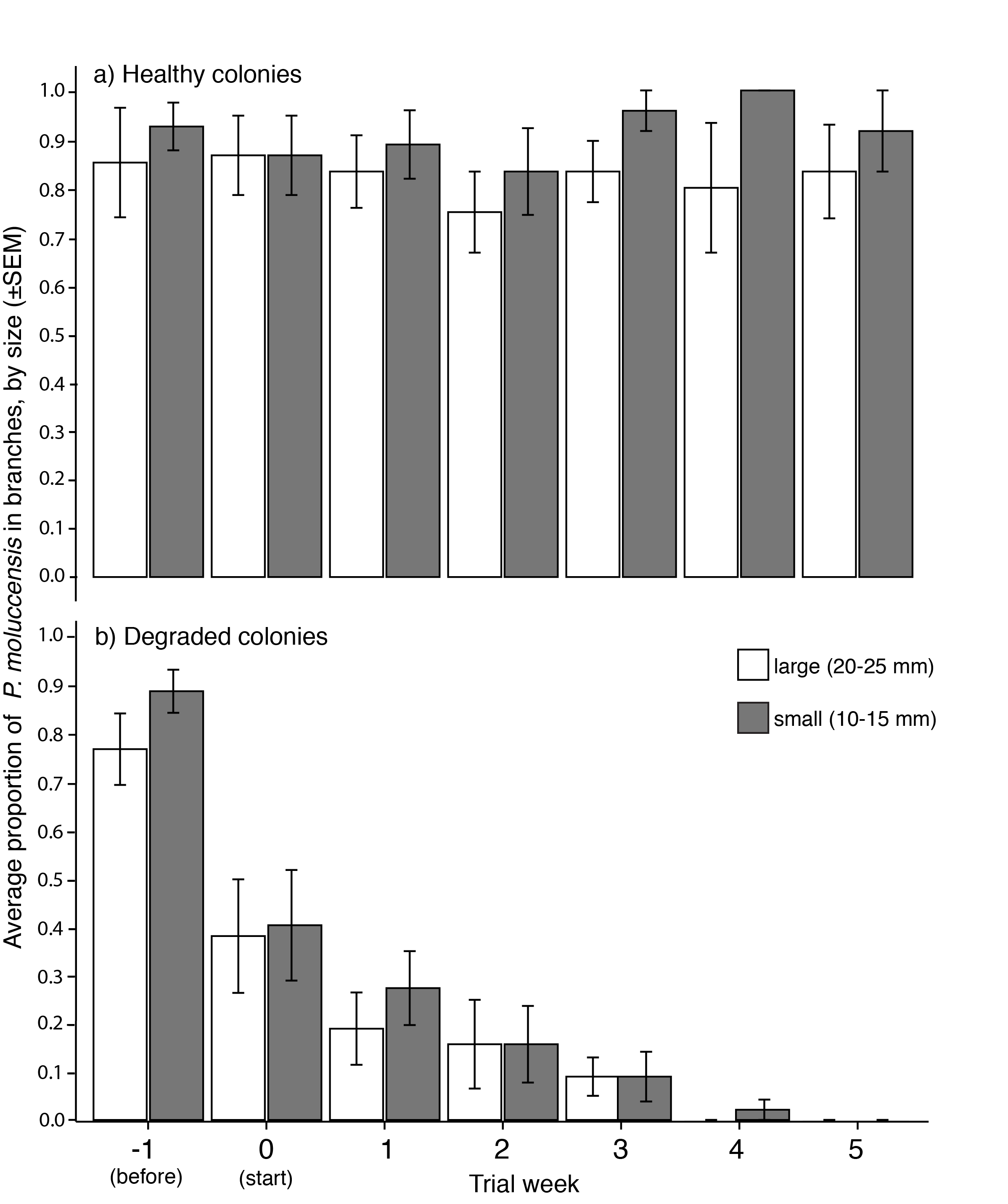


Supplemental Figure 2: Comparison of the average proportion (out of 3 in each size class per trial) of large (20-25mm, white bars) and small (10-15mm, grey bars) *P. moluccensis* sheltering within the coral branches on control (a, live) and treatment (b, degraded) over seven trial weeks. Treatment colonies gradually accumulated algae, sponges and settling invertebrates during the five weeks following the death of 100% of the colony tissue. Error bars indicates SEM.
